# Working with women to improve child and community eye health

**Published:** 2009-06

**Authors:** Gopa Kothari

**Affiliations:** Managing Trustee, Child Eye Care Charitable Trust and Director: Community Programmes, Operation Eyesight, Kusum Kunj, 3rd Floor, 10th Road, Khar West, Mumbai 400052, India. Email: gkothari.cecct@gmail.com and gopajay@hotmail.com

**Figure:**
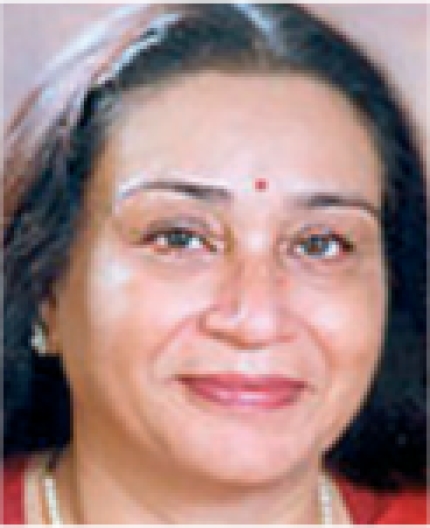


In the slums and rural areas of India, visual impairment, blindness, and childhood blindness are usually more prevalent.

In order to improve the eye health of children and the community in these areas, it is important to understand the influence women and mothers have over children's eye health and the eye health of the community as a whole.

## The social context

In these slums and rural areas, most families are poor. Women also tend to have lower levels of education, less financial independence, and lower social status than men. But why is this so?

There is also a strong preference for sons in these areas of India, with daughters being viewed as economic burdens. This preference for sons, combined with high dowry costs for daughters, means that many parents would rather pay for their sons than their daughters to receive education or vocational training - especially when families are poor and their resources are limited.

When daughters reach adulthood, they are less educated than their brothers and therefore less able to earn a living and to be financially independent. This reinforces the view of women as economic burdens and contributes to their lower social status.

As a result, women often have very little say in how family resources are allocated are less able to ensure that their children, daughters in particular, will receive the eye care they need. They also have less influence in community decisions that affect eye health.

## The importance of education

Researchers have recognised that educating girls is important for improving health, reducing gender inequality, and empowering women.^1^ Every extra year of maternal education in low- and middle-income countries reduces under five child mortality by 5–10 per cent. Educated women are also more likely to make use of health services, including eye care services, for their children.^2^

Education improves health outcomes by increasing women's ability to acquire and use health-related information and services.^3^ Education and the resulting economic independence and increased status of women also gives greater bargaining power in household decisions and personal relationships, which often translates into increased allocation of household resources to child health and nutrition.^4^,^5^

**Figure:**
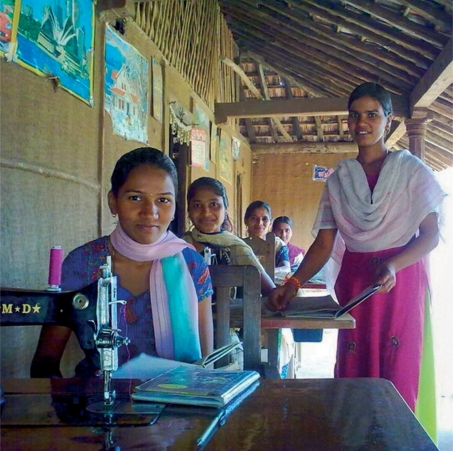
Women in Dang are trained in tailoring. INDIA

Shilpa Vinod Bhatte

## Addressing the problem

Although it would be ideal to ensure that girls received the same levels of education as boys, when this is not possible we can still improve the situation of adult women by:

- providing health education which women can use to improve their family's eye health
- providing literacy and vocational skills training to allow women to improve their financial independence and, as a result, their influence in family decisions.

Ultimately, the community as a whole will benefit: when women have higher social status, they are in a better position to help improve conditions in their community that will lead to better eye health. These include provision of safe drinking water, sewage disposal, toilet facilities, and sanitation within accessible reach of households. Women have an important role to play in the planning, delivery, and maintenance of such services.

## Health education

Health education for women should include:

- information about child rearing and feeding practices
- basic eye care and hygiene
- the causes and treatment of common eye diseases
- prevention of eye injuries
- basic first aid
- advice about when to seek professional care.

Targeting mothers in the antenatal and postnatal period helps in the prevention of ophthalmia neonatorum and other eye infections due to harmful traditional practices. Vitamin A deficiency can be prevented by informing mothers about the importance of vitamin A-rich foods and exclusive breastfeeding. Mothers should also receive information about the importance of primary immunisations such as measles, as these will help prevent nutritional blindness.

## Literacy and skills training

Literacy and skills training for women and mothers can improve their confidence and ability to function in society as well as their ability to earn income for their family. Being literate not only helps women to access health information and information about available eye care services, but also enables mothers to educate their children with regards to eye care and eye hygiene.

**Figure:**
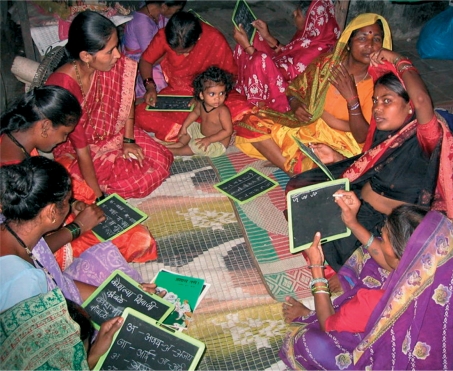
Women in a slum gather at an adult literacy class in the community. INDIA

Child Eye Care Charitable Trust

Learning new skills can enable women to start their own small businesses and earn an income. In addition, teaching women how to use micro-financing and other saving schemes will help them make the most of the money they earn. As income earners, women not only help to improve their family's ability to afford better food, eye care, and education for their children, they also enjoy better social status within the family and community.^6^

## Women's groups (self-help groups)

The author's non-profit organisation, Child Eye Care Charitable Trust (CECCT), runs holistic community eye care programmes in slums in Mumbai and rural communities in the Dang tribal area in Gujarat. These programmes, which are sponsored by Operation Eyesight, aim to meet a community's need not only for good vision, but also for health, nutrition, education, and economic independence.

Women's groups, also known as self-help groups, are an important component of the trust's work. They are a means to provide health education as well as literacy and skills training to women in poor communities. Groups consist of fifteen to twenty women from the same area who meet once or twice a month.

With the guidance of the trust and local non-government organisations, the women receive training in skills such as making candles, soap, chalk, incense sticks, artificial flowers, or pickles. These skills can usually be acquired within one to three months and enable women to start their own small business.

Women are also taught how to set up a bank account in which they deposit their monthly income. When the women in the group have deposited a set amount per month, they can then apply to the bank for registration, which entitles them to receive credit from the bank and concessions in various government schemes, meant specifically for women from poor families.

The groups receive adult literacy training so that they are able to read and write and maintain their own bank accounts and savings. In the tribal areas, the trust collaborates with other organisations to teach women agricultural skills; this helps to improve their food security.

In the groups, women also learn eye care and health awareness messages which they in turn convey to their families and the rest of the community. Women who are very motivated and have good communication skills are trained as primary eye care workers or community health workers. Primary eye care workers are able to treat people at the community level or refer them to the nearest base hospital. Community health workers are trained to provide care that is complementary to eye care services, such as nutrition education, health education, and so on. Both types of workers are able to detect eye health problems at the community level.

Since these workers are from the community itself, their services are readily available, accessible, and acceptable to other women; this helps to fight gender inequalities in providing eye care services to those women who usually do not seek eye care for themselves or their children, especially their daughters.

Recently, the trust has started to send a small number of men and women from both Mumbai and Dang to train as vision technicians at LV Prasad Eye Institute. This gives community health workers and primary eye care workers the opportunity to take on a larger and more specialised role in eye care. The trust aims to establish vision centres, staffed by these vision technicians, in each area so that good quality eye care services are available within the community itself.

**What can we do?**

- Talk to women and mothers about the eye health of their children. Encourage them to bring children for treatment and help them understand what they can do to improve the likelihood that their children will have good vision.
- Support initiatives to improve literacy among women and to train them in income-generating activities.
- Target health education at different generations: include grandmothers (through ‘granny groups’) as well as children. Role playing and stories can be used to teach children to adopt positive eye care practices which they will pass on to other children and to their families.
- Involve key community leaders and representatives of self-help groups when designing projects to create awareness of eye care.
- Take care not to exclude men. Involvement of women in income-generating activities, for example, can result in men feeling excluded and resentful, especially when projects focus only on women. It is important to include men as doing so will ultimately benefit the family and also prevent feelings of exclusion and resentment.
